# Lysosomal dysfunction–induced autophagic stress in diabetic kidney disease

**DOI:** 10.1111/jcmm.15301

**Published:** 2020-06-25

**Authors:** Hui Juan Zheng, Xueqin Zhang, Jing Guo, Wenting Zhang, Sinan Ai, Fan Zhang, Yaoxian Wang, Wei Jing Liu

**Affiliations:** ^1^ Renal Research Institution of Beijing University of Chinese Medicine Beijing China; ^2^ Key Laboratory of Chinese Internal Medicine of Ministry of Education and Beijing Dongzhimen Hospital Affiliated to Beijing University of Chinese Medicine Beijing China; ^3^ Institute of Nephrology, and Zhanjiang Key Laboratory of Prevention and Management of Chronic Kidney Disease Guangdong Medical University Zhanjiang China

**Keywords:** autophagic stress, autophagy, diabetic kidney disease, lysosomal dysfunction

## Abstract

The catabolic process that delivers cytoplasmic constituents to the lysosome for degradation, known as autophagy, is thought to act as a cytoprotective mechanism in response to stress or as a pathogenic process contributing towards cell death. Animal and human studies have shown that autophagy is substantially dysregulated in renal cells in diabetes, suggesting that activating autophagy could be a therapeutic intervention. However, under prolonged hyperglycaemia with impaired lysosome function, increased autophagy induction that exceeds the degradative capacity in cells could contribute toward autophagic stress or even the stagnation of autophagy, leading to renal cytotoxicity. Since lysosomal function is likely key to linking the dual cytoprotective and cytotoxic actions of autophagy, it is important to develop novel pharmacological agents that improve lysosomal function and restore autophagic flux. In this review, we first provide an overview of the autophagic‐lysosomal pathway, particularly focusing on stages of lysosomal degradation during autophagy. Then, we discuss the role of adaptive autophagy and autophagic stress based on lysosomal function. More importantly, we focus on the role of autophagic stress induced by lysosomal dysfunction according to the pathogenic factors (including high glucose, advanced glycation end products (AGEs), urinary protein, excessive reactive oxygen species (ROS) and lipid overload) in diabetic kidney disease (DKD), respectively. Finally, therapeutic possibilities aimed at lysosomal restoration in DKD are introduced.

## 1 INTRODUCTION

Diabetic kidney disease (DKD) is the most common microvascular complication of diabetes and the leading cause of end‐stage renal disease worldwide.^1^ Due to its rapidly increasing prevalence, poor prognosis and limited treatment options, DKD has become an urgent and important public health concern.^2^ The development of kidney damage in diabetic patients is the result of multifactorial interactions between intracellular metabolic and hemodynamic abnormalities, involving prolonged hyperglycaemia, advanced glycation end products (AGEs) accumulation, reactive oxygen species (ROS) overproduction, massive proteinuria and lipid overload.^3^, ^4^, ^5^ The pathological alterations of DKD implicate virtually all of the renal intrinsic cells; for example, podocytes are terminally differentiated glomerular epithelial cells with limited capacity to proliferate in vivo; therefore, the injury and loss of podocytes are particularly vulnerable to impairment of clearance during DKD progression.^6^ Similarly, proximal tubular epithelial cells (PTECs) are exposed to various toxic factors in the glomerular filtrate due to their major reabsorptive function within the nephron, which are associated with tubular hypertrophy and progressive interstitial fibrosis.^7^ Moreover, in response to injury and progressive DKD, mesangial cells proliferate and produce excessive extracellular matrix (ECM) in the mesangium, contributing to glomerulosclerosis and renal fibrosis.^8^ Thus, the intracellular degradation system is believed to be crucial for maintaining renal cell homeostasis and function in diabetes.

Macroautophagy, hereafter termed as autophagy, is a major intracellular degradation pathway that maintains intracellular integrity by delivering cytoplasmic materials to lysosomes for digestion and recycling. A growing body of evidence has suggested that autophagy is defective in diabetic kidneys and that its activation serves as a protective response, suggesting that autophagy may be a promising therapeutic target for DKD.^9^, ^10^ Previous studies have revealed that autophagy‐deficient mice fail to up‐regulate lysosomal biogenesis, leading to AGE accumulation in renal cells and thus kidney injury.^11^ Autophagy also plays a crucial role in maintaining lysosomal homeostasis, with its impairment in DKD resulting in the accumulation of damaged lysosomes followed by podocyte apoptosis.^12^ These studies demonstrate the importance of autophagy induction in maintaining lysosomal function and protecting nephrons against injury during diabetes. However, the autophagic process is a very complicated pathway, involving autophagic vacuole formation, fusion between autophagic vacuoles and lysosomes, and the lysosomal degradation of autophagic vacuoles; therefore, damage or blockade of any node inactivates autophagy. Lysosomes, which are the major degradative compartment, are located at the terminal process of the autophagic pathway and play a crucial role in the autophagic degradation of macromolecules and organelles.^13^ Several studies have shown that diabetes can reduce enzyme activity and albumin degradation in lysosomes.^14^, ^15^ The levels of lysosomal transcription factor were also observed to be reduced in the kidneys of DKD patients.^16^ Indeed, aetiological factors in DKD may trigger lysosomal dysfunction, which results in blockage of the downstream pathway of the autophagic process,^17^, ^18^ and thereby contributing to a condition of autophagic stress in which rates of autophagic vacuole formation exceed rates of its degradation.^19^ Basal or increased levels of autophagy induction in cells with pathologically impaired lysosomal function can cause cellular injury and even death, which may be implicated in the development and progression of DKD.^19^, ^20^ Thus, it is essential to understand the exact start point and key node of the defective autophagic‐lysosomal process, and identify therapies that restore lysosomal function and then relieve autophagic stress for the treatment of DKD.

Herein, we aim to review the role of autophagic stress due to lysosomal dysfunction in the pathogenesis of DKD, which consequently opens the path to the discovery of novel therapeutic strategies for DKD.

## 2 THE AUTOPHAGIC‐LYSOSOMAL PATHWAY

**FIGURE 1:**
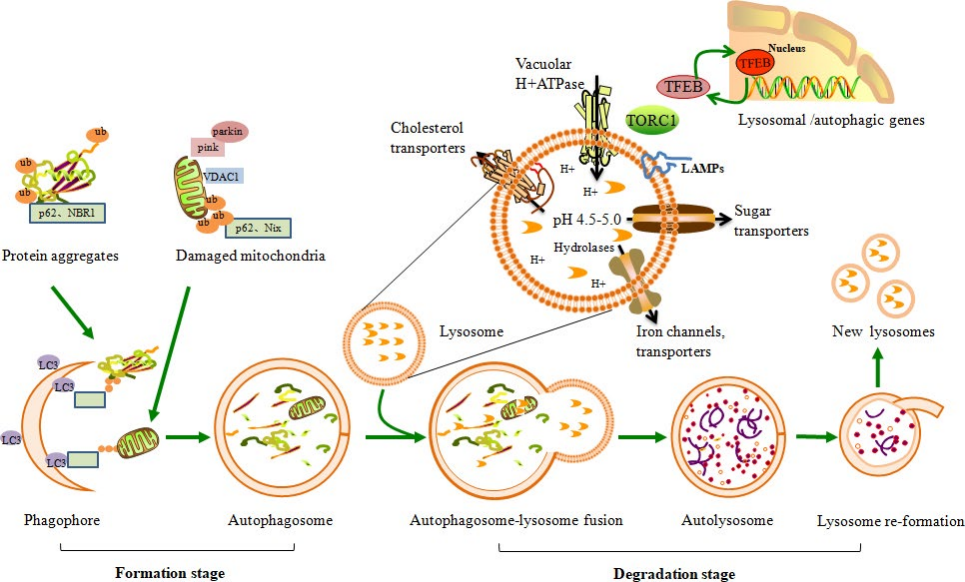
Schematic depiction of the autophagic‐lysosome pathway. Protein aggregates and damaged mitochondria are selectively targeted for autophagy via ubiquitin (Ub)‐labelled adaptor proteins (eg p62, Nix and NBR1). Autophagy occurs when the autophagy receptors bind cargoes with LC3 to initiate substrate sequestration in the forming autophagosome. Matured autophagosomes fuse with lysosomes containing hydrolytic enzymes for cargo degradation. When the degradation is complete inside the autolysosome, the lysosomes regain their original properties via lysosome re‐formation. The lysosome comprises a specific set of luminal and integral‐membrane proteins. The acidification of the lysosome is maintained by the vacuolar‐H+ ATPase and the lysosomal membrane, which contains highly glycosylated lysosome‐associated membrane proteins (LAMPs), ion channels and transporters, and cholesterol transporters. Moreover, the biogenesis and function of lysosomes are also regulated by transcription factors such as TFEB. As terminal degradation stations, normal morphology and function of lysosomes are essential for successful completion of autophagic degradation

Autophagy is an intracellular process that involving the lysosomal degradation and recycling of unwanted cytoplasmic proteins and organelles. Three types of autophagy have been characterized in mammalian cells, including macroautophagy, chaperone‐mediated autophagy and microautophagy. Macroautophagy is, by far, the most intensively studied in DKD and is the focus of this review. In autophagy, the autophagosomes recognize and sequester intracellular cargo tagged by autophagic receptors/adaptors, such as sequestosome 1(p62/SQSTM1), neighbour of Brca (NBR1) and Nix.^21^, ^22^ In addition to the bulk degradation of cytoplasmic contents, autophagy can be specific due to cargo selectivity, such as aggrephagy (protein aggresomes),^23^ mitophagy (damaged mitochondria),^24^ lysophagy (damaged lysosomes)^25^ and lipophagy (lipid droplets).^26^ Autophagy is a highly dynamic and multi‐step process often referred to as autophagic flux (Figure 1) and can be constitutive or inducible, enabling the cell to rapidly adapt to altered intracellular and extracellular environments. Maintaining efficient autophagic flux requires the co‐ordination of a suite of autophagy‐related genes (Atg), lysosomal proteins and phospholipids.^27^, ^28^ Genetic Atg deletions and pharmacological autophagy inhibitors have revealed the crucial role of autophagy in clearing macromolecular proteins and damaged organelles.^12^, ^29^ Dysregulation of the autophagic process may be involved in the pathogenesis of DKD.

### 2.1 The formation stage

Metabolic stress can initiate phagophore formation mediated by ULK1 and class III phosphatidylinositol 3‐kinase (PI3K) Vps34 complexes that control the recruitment of specific proteins into newly forming autophagosomal membranes. Isolation membrane (phagophore) elongation and autophagosome closure require two ubiquitin‐like conjugating systems, namely the Atg12‐Atg5‐Atg16L and microtubule‐associated protein 1 light chain 3 (LC3) complexes.^30^ The cytosolic LC3‐I covalently conjugates with phosphatidylethanolamine (PE) to form the lipidated compound LC3‐II on autophagosomal membranes. Since LC3 levels are associated with autophagosome number and are considered a marker of autophagosome formation, LC3‐based assays are widely used to assess autophagosome formation and autophagic flux when autophagosome‐lysosome fusion is hampered.^31^ In addition, degradation of the ubiquitin‐binding protein p62/SQSTM1, an adaptor that links autophagy‐defined structures to autophagosomes, is often considered a marker of autophagic flux.^31^, ^32^

### 2.2 The degradation stage

#### 2.2.1 Fusion of autophagosomes with lysosomes

Once membrane closure is complete, the autophagosome subsequently fuses with lysosomes to form the autolysosome. The process of autophagosome‐lysosome fusion needs to be co‐ordinated to guarantee successful delivery of the engulfed contents to lysosomes for degradation. Lysosomes contribute a subset of soluble NSF attachment protein receptors (SNAREs) involved in fusion.^33^ Furthermore, lysosomes provide the membrane‐localized Rab proteins and lysosome‐associated membrane proteins (LAMPs) collectively aid in the fusion events. Among them, interactions of Rab7 and other endolysosomal proteins influence lysosome positioning.^34^ The lysosome‐tethered homotypic fusion and vacuolar protein (HOPS) tethering complex bring together the lysosomal and autophagosomal membranes.^35^ Breakdown of the inner autophagosomal membrane initiates the process of autophagosomal cargo degradation.

#### 2.2.2 Lysosome degradative machinery

Upon fusion with autophagosomes, mature lysosomes with a single‐lipid bilayer and highly acidic milieu (pH ~ 4.5) introduce the necessary degradative machinery, including up to 60 different acid hydrolases, such as proteases, nucleases, glycosidases and lipases to degrade macromolecules.^36^, ^37^ Luminal acidification of lysosomes is highly important and is maintained by vacuolar‐type H+‐ATPase (v‐ATPase), a large protein complex on the lysosomal membrane that pumps protons into the lysosomal lumen.^38^ Indeed, luminal alkalinization by v‐ATPase inhibitors such as bafilomycin A1^39^ and lysosomotropic basic amphiphiles such as chloroquine^40^ can halt digestion, while lysosomal cathepsin (B, L and D) deficiency prevents protein degradation and leads to the accumulation of undigested cargo.^41^, ^42^ In addition, the lysosomal membrane comprises highly glycosylated LAMPs that protect the lysosomal membrane from self‐digestion, ion channels and transporters that maintain ion homeostasis, cholesterol and other lipid transporters that participate in the export of sugars, amino acids and other products of lysosomal degradation.^43^ However, metabolic stress, cholesterol and urate crystals can damage lysosome integrity by inducing lysosomal membrane permeabilization (LMP) or rupture, resulting in cathepsin leakage and programmed cell death.^44^, ^45^

#### 2.2.3 Lysosome biogenesis

Lysosome biogenesis is co‐ordinated by transcription factors in the microphthalmia‐transcription factor E (MiT) family, including transcription factor EB (TFEB) and TFE3.^46^ TFEB is a master regulator of co‐ordinated lysosomal expression and regulation (CLEAR) that up‐regulates most genes involved in lysosomal biogenesis and those required for autophagosome formation.^46^, ^47^ TFEB nuclear translocation is promoted by diverse environmental cues such as starvation, oxidative stress and mitochondrial damage, which allow metabolism and adaptation of lysosome to cellular needs.^48^ Under nutrient‐scarce conditions, inhibited mammalian/mechanistic target of rapamycin (mTOR) complex 1 (mTORC1) relocates away from the lysosomes, inducing autophagy and allowing the nuclear translocation of dephosphorylated TFEB, thereby promoting the transcription of the lysosomal genes. In contrast, in nutrient‐rich conditions, TFEB is recruited to the lysosomes, phosphorylated by mTORC1 and retained in the cytoplasm by binding 14‐3‐3 proteins.^49^ Thus, the up‐regulation of TFEB expression increases the number of lysosomes by promoting the biogenesis of new lysosomes. In addition, TFE3 also regulates lysosome biogenesis and functions at the gene expression level, and evidence has shown that TFE3 plays a cooperative role with TFEB in the regulation of glycolipid metabolism.^50^ Similarly, pH, nutrients and cellular stress can activate the transient receptor potential cation channels of the mucolipin family (TRPML) of lysosomal Ca^2+^ channel by mediating Ca^2+^ release from the lysosomal lumen into the cytosol. TRPML1 acts in a positive feedback loop with TFEB that promotes intracellular clearance of accumulating substrates.^51^

#### 2.2.4 Lysosome re‐formation

Degradation products such as amino acids and sugars are transported out of the autolysosomes via a family of lysosome efflux transporters, and autolysosomes disintegrate once autophagy is terminated. Lysosomes regain their original identity via mTORC‐mediated autophagic‐lysosome re‐formation (ALR).^52^ After prolonged starvation, mTOR is reactivated to attenuate autophagy and generate vesicles from autolysosomes that ultimately mature into functional lysosomes.^53^ Thus, this process helps to restore a full complement of lysosomes. As the constituents of the autophagic machinery are consumed during autophagy, these must be resynthesized during stress to sustain autophagic flux. Thus, as the end‐point of the autophagic trafficking pathway, the lysosome is an important target for regulating cellular autophagy.

## 3 ADAPTIVE AUTOPHAGY AND AUTOPHAGIC STRESS

**FIGURE 2:**
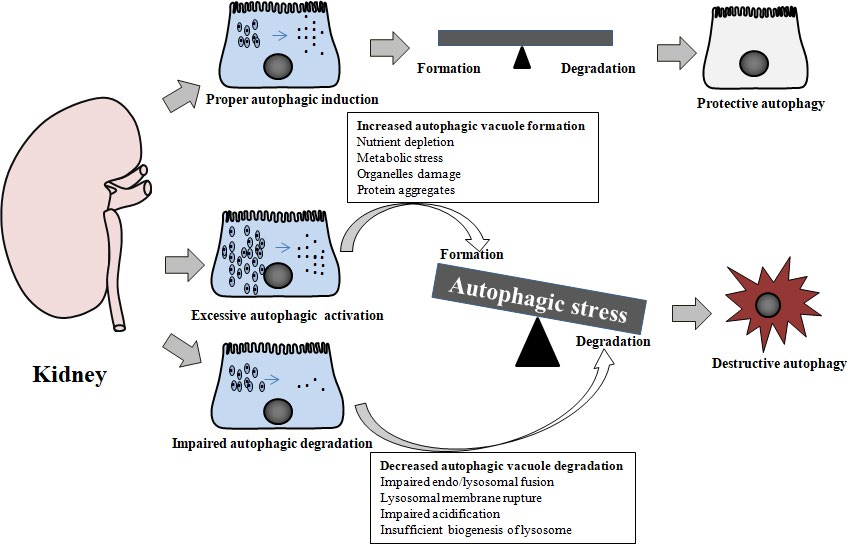
Schematic diagram displaying the dual roles of autophagy based on lysosomal function in kidney. When lysosome function is normal, proper autophagy induction in response to stress factors maintains or restores cellular homeostasis to protect against cell injury and thus promotes cell survival in the kidney. Prolonged and intense autophagy induction in response to damaged cellular constituents exceeding the degradative capacity of lysosomes or normal autophagy induction accompanied by marked impairment of lysosomal degradation, result in autophagic stress. The condition of autophagic stress contributes to aggravated cell damage and/or autophagic cell death in kidneys

Several stimuli promote autophagy representing an essential mechanism by which cells can adapt to stress conditions.^54^ Autophagy‐related renoprotective mechanisms against kidney injury have been demonstrated by pharmacological or genetic autophagy inhibition, which reduce the ability of kidneys to respond to various pathogenic factors.^55^, ^56^ However, when lysosomal degradation is limited, basic or robust autophagic responses are futile and wasteful to cells. Over time, stress factors are likely to cause a feedback loop inducing more autophagic vacuole formation, thereby further aggravating lysosome injury and triggering cell death.^57^ Thus, these apparently conflicting roles of autophagy‐related processes in both adaptive/protective and detrimental/death‐associated pathways have been identified in some nephropathies,^58^ which are likely due to differences between proper autophagic activation and increased autophagic stress,^59^, ^60^ as shown in Figure 2. From this perspective, lysosomal function may be the key to link the dual actions of autophagy, since autophagy‐dependent adaptive responses are mounted under normal lysosome conditions yet autophagic cell death is triggered by abnormal lysosomes.

### 3.1 Proper autophagy activation has pro‐survival effects in normal lysosomal conditions

Autophagy plays a housekeeping roles by degrading aggregate‐prone proteins, damaged organelles and other macromolecules, but can also act as an adaptive mechanism in response to cellular stresses, such as hypoxia, oxidative stress and endoplasmic reticulum stress to maintain cell homeostasis and promote cell survival.^61^ Autophagy is also regulated by the nutrient‐sensing pathways via mTOR, sirtuins (SIRT) and AMP‐activated protein kinase (AMPK).^9^ Autophagy‐dependent processes promote cellular adaptation and restore cellular homeostasis in different cell types through a common mechanism, namely the clearance of protein aggregates and unwanted organelles, which prevent inflammation, oxidative stress and apoptosis.^62^ When this adaptive attempt fails, damaged organelles and ubiquitinated proteins may accumulate in renal cells and exacerbate DKD.^12^, ^17^ Thus, proper ‘physiological’ autophagy activation in response to multiple pathogenic injuries promotes cell survival under normal lysosomal conditions.

### 3.2 Increased or normal autophagy triggers autophagic stress and subsequent cellular injury when lysosomal function is impaired

Mechanisms that promote the autophagic degradation of damaged organelles and protein aggregates are likely only effective if autophagic degradation can be completed. Altered lysosomal function may reduce the efficacy of normal or increased autophagic responses that remove cytoplasmic cargoes, instead creating autophagic stress in response to additional stresses.^60^ The abnormal state of lysosomes, including changes in lysosomal membrane integrity, volume, enzymatic activities and failure to generate new functional lysosomes, gives rise to a blockage of the autophagic process, which has been implicated in DKD.

#### 3.2.1 Degradative dysfunction of ‘old lysosomes’ due to changes in lysosomal membranes and enzymes

Numerous endogenous and exogenous stimuli, such as excessive ROS or treatment with lysosomotropic agents may damage lysosomes by inducing LMP.^63^, ^64^ This lysosomal damage causes hydrolytic enzymes to leak into the cytoplasm and eventually triggers a cascade of proteolysis of cellular organelles that often results in apoptosis or necrosis.^45^ In addition, an increase in lysosome size and volume may be a result of cellular injury during sustained high requirement for hydrolysis, indicating potential lysosomal damage.^65^, ^66^ Similarly, increased lysosomal pH could impair the enzymatic activity and decrease the acidification function of lysosomes.^67^ Functional lysosomes progressively deteriorate with age while accumulating lipofuscin and protein aggregates and declining functionally, which is directly responsible for impaired autophagy and accelerated cellular senescence in diabetic kidneys.^68^, ^69^ As observed in diabetes mellitus, a representative of age‐related diseases, blockage of the autophagy‐lysosome system has been associated with premature ageing of kidneys.^29^, ^70^ Furthermore, ‘old’ lysosomes may be dysfunctional respond to increased or continued degradation requirements. Damaged lysosomes can be selectively sequestered by autophagic vacuoles during lysophagy, which is likely a typical feature of increased autophagic stress as it suggests the apparent formation of autophagosomes and impairment of lysosomal degradative capacity.^71^, ^72^

#### 3.2.2 Insufficient number of ‘new lysosomes’ results from disruption of lysosomal biogenesis

The subcellular localization of TFEB is dependent on nutritional or environmental cues. Some stress conditions, such as oxidative stress, inflammation, endoplasmic reticulum stress and mitochondrial damage, activate mTORC1‐mediated phosphorylation that inhibits TFEB nuclear translocation.^73^, ^74^, ^75^ Such cytoplasmic retention of TFEB is associated with decreased lysosomal biogenesis that results in a reduced number of functional lysosomes for autophagic clearance. Conversely, several studies have shown induction of lysosomal biogenesis by overexpression of TFEB results in intracellular clearance of undegraded substrates and subsequent attenuating pathological manifestation.^76^, ^77^ Furthermore, as lysosomes are continuous consumed by hybrid organelles, lysosome regeneration forms new functional lysosomes that maintain lysosome homeostasis and autophagic flux. It seems reasonable to suspect that the pathophysiological conditions in kidney diseases can elicit similar lysosome‐mTORC1 inter‐relationships.^78^ ALR defects or impaired lysosome recycling may decrease the number of newly generated lysosomes in vivo, causing inefficient autophagic clearance and the accumulation of undegraded cellular proteins.^79^ Additionally, defects in genetic factors that encode lysosome‐related genes also reduce the number of functional lysosomes; for instance, deficiency of cystinosin reduced TFEB expression and induced TFEB nuclear translocation, leading to the accumulation of non‐degraded materials in lysosomes similar to that which occurs in lysosomal storage diseases.^80^ Together, transcriptional defect in lysosomal genes inhibits the biogenesis of new functional lysosomes, resulting in the insufficiency of lysosomes to complement the consumption during the autophagic process. When sustained autophagic demand cannot be balanced by the lysosomal degradative systems, it may render renal cells particularly susceptible to autophagic stress.

## 4 AUTOPHAGIC STRESS IN DKD

**FIGURE 3:**
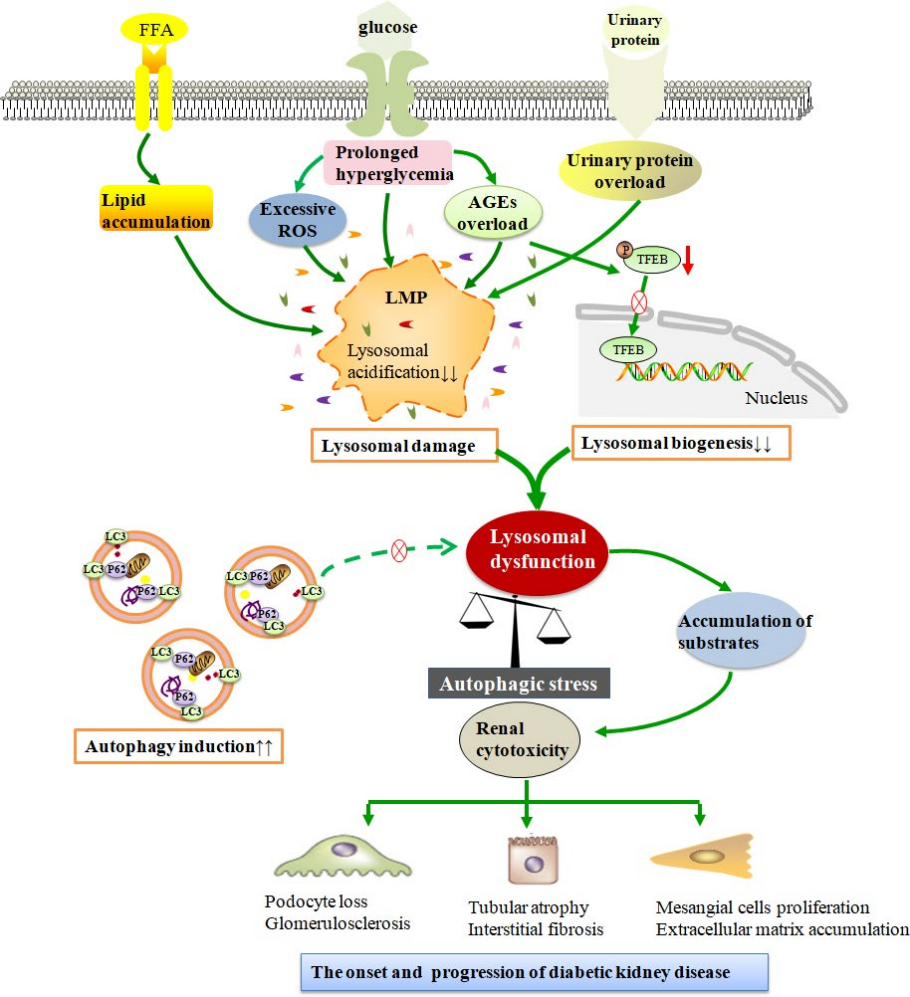
Schematic diagram displaying the role of autophagic stress in various pathogenic factors for diabetic kidney disease. In a chronic diabetic state, various pathogenic factors of diabetic kidney disease, such as prolonged hyperglycaemia, advanced glycosylation end products (AGEs) overload, urinary protein overload, lipid droplets accumulation and excessive reactive oxygen species (ROS), trigger lysosomal dysfunction due to lysosomal membrane permeabilization (LMP) and/or reduced lysosomal biogenesis. Under these conditions, increased or sustained demand for autophagy induction in cells accompanied by the impaired clearance of cellular components promotes autophagic stress, resulting in the accumulation of damaged organelles and/or abnormal proteins in renal cells. The condition of autophagic stress in renal cells causes a switch from a renoprotective mechanism to a cytotoxic state, which is implicated in the pathogenesis of diabetic kidney disease

Recently, accumulating evidence has demonstrated that impaired autophagy is involved in the pathogenesis of DKD, suggesting that autophagy activation could be a therapeutic target for DKD. Studies from our group and others have found that lysosomal membrane damage and lysosomal dysfunction may be triggered in podocytes and PTECs under DKD conditions, accompanied by accumulation of the p62 and ubiquitinated proteins.^12^, ^17^ Several studies have also shown that diabetes and the associated long‐term metabolic alterations could cause a decrease in lysosomal enzyme activity.^16^, ^81^ Moreover, TFEB mRNA and protein levels were observed to be decreased in PTECs of DKD patients.^16^ Although autophagic degradation is reduced in diabetic kidneys, the need for protective autophagy is significantly increased due to greater exposure to cellular stresses. Increased autophagy induction alongside impaired degradation of autophagic vacuoles creates autophagic stress that may contribute to kidney injury in DKD.^19^ Since lysosomal function is the main determinant of adaptive autophagy or autophagic stress, it is important to understand how autophagy is altered by each aetiological factor in DKD to target it therapeutically (Figure 3).

### 4.1 Time‐dependent hyperglycaemia intervention plays dual roles in autophagy

In diabetes, metabolic factors such as insulin, metabolites, growth factors and hormones affect autophagic activity, with in vivo flux assays using chloroquine suggesting that autophagy is substantially activated in the renal cells of streptozotocin (STZ)‐treated mice.^55^, ^82^ Mounting evidence has highlighted the renoprotective role of an intact autophagic flux against high glucose–induced renal injury. Basal levels of autophagic flux are high in differentiated podocytes; however, interruption of this flux results in a dramatic increase in susceptibility to podocyte loss and glomerulosclerosis,^83^ highlighting the importance of autophagy as a defence mechanism for maintaining glomerular homeostasis. Although autophagy activity is relatively low under basal conditions, higher rates of autophagy are essential for PTECs under stress conditions. Indeed, its inhibition with pharmacological inhibitors (3‐methyladenine or CQ) or Atg5 knockdown in high glucose–treated PTECs exacerbates kidney injury, suggesting that autophagy may protect against hyperglycaemia‐induced tubular cell injury.^84^ Thus, these findings indicate that autophagy induction is a predominantly an adaptive renoprotective process that removes damaged organelles and protein aggregates in diabetic kidneys.

The effect of high glucose levels on autophagy appears to be time‐dependent. A STZ‐induced diabetic model revealed stronger GFP‐LC3 staining and LC3B‐II turnover in glomeruli after four weeks, suggesting that hyperglycaemia induces autophagic flux in glomeruli in early‐stage diabetes. Conversely, LC3 staining was reduced, along with p62/SQSTM1 accumulation in the cytoplasm of glomerular cells after eight weeks, confirming a disruption of autophagic flux in glomeruli under long‐term hyperglycaemia.^55^ Consistently, autophagy is increased in high glucose–cultured PTECs after 6 and 12 hours but repressed after 48 or 96 hours, suggesting that hyperglycaemia‐induced intracellular stress may activate autophagy in response to short‐term high‐glucose exposure.^84^ Under prolonged hyperglycaemia, sustained disturbance to nutrient‐sensing pathways could impair the autophagic response in the kidney, resulting in the accumulation of misfolded proteins and damaged organelles in renal cells and the acceleration of pathological DKD progression.

The mechanisms underlying duration‐related autophagy inactivation under high glucose conditions may be associated with impaired lysosomal function. Previous studies have revealed that diabetes can reduce the activity of lysosomal cathepsins, indicating reduced lysosomal degradation in isolated glomeruli or renal tubular in diabetes.^16^, ^85^, ^86^ Moreover, studies have revealed that autophagy induction plays a critical role in maintaining lysosomal biogenesis and function in PTECs. For instance, prolonged high‐glucose exposure reduces lysosomal activity (as assessed by cathepsin D maturation) and deforms lysosomes in PTECs.^87^ In addition, mTORC1 is hyperactivated by multiple extra‐ and intracellular cues under hyperglycaemic conditions, in turn inhibiting autophagy induction by phosphorylating ULK1 and inhibiting TFEB nuclear translocation to suppress lysosomal biogenesis and autophagy.^88^, ^89^ Other kinases, such as AMPK and Akt (protein kinase B) also phosphorylate MiT‐TFEs to affect their subcellular localization and lysosomal nutrient‐sensing under diabetic conditions.^90^ Thus, sustained hyperglycaemic exposure may gradually impair lysosomal function and blunt the lysosomal degradation of autophagic vacuoles. In diabetic kidneys, not only is lysosomal degradation reduced but also the need for autophagy induction is increased due to high exposure to cellular stresses in DKD. This increased or continued demand for autophagy in the face of impaired degradative efficiency contributes to autophagic stress in the diabetic kidney. Further research is required to elucidate the mechanisms underlying lysosomal dysfunction in hyperglycaemia conditions.

### 4.2 AGEs trigger autophagic stress by blocking lysosomal degradation

AGEs are formed by the irreversible attachment of reducing sugars onto protein amino groups under long‐term hyperglycaemia. Chronic AGE accumulation amplifies hyperglycaemic stress, initiating a vicious cycle of metabolic disturbance.^91^ Exogenous AGEs from the circulation or glomeruli filtrate are endocytosed alongside endogenous AGEs generated by the lysosomal degradation of glycated proteins in PTECs. Emerging evidence has demonstrated that AGE overload gradually disrupts autophagic flux, as evidenced by decreased Lamp1 transcription.^11^ AGE accumulates in autophagy‐deficient kidneys due to insufficient lysosomal degradation in the proximal tubules. We and other groups have shown that AGEs impair autophagy in podocytes and PTECs, and the number of autolysosomes decreases with AGEs stimulation.^17^, ^18^ Our studies have also revealed that AGEs induce stagnation of the autophagy‐lysosomal pathway due to LMP and defective lysosome acidification, causing accumulation of abnormal proteins. Similarly, in long‐term AGE‐stimulated endothelial cells, impaired autophagic flux with blockage of autophagosome‐lysosomal fusion leads to apoptosis in diabetes.^92^ AGEs place huge stress on lysosomes to blunt autophagic flux, with high‐AGE conditions increasing the number of autophagosomes but decreasing autolysosomes to cause autophagic stress, which in turn contribute to cytotoxicity in the diabetic kidney.^19^

Many studies have investigated the mechanisms underlying AGE‐mediated lysosomal impairment. In diabetes, increased AGE levels activate the AGE receptor (RAGE), which mediates kidney injury in diabetic models. RAGE Activation directly induces ROS production via NADPH oxidase and establishes redox crosstalk with mitochondria that amplifies ROS generation.^93^ Consistently, we found that the AGEs‐RAGE axis evokes oxidative stress, triggers LMP and plays a critical role in lysosomal dysfunction.^17^ Thus, antioxidants may improve lysosomal dysfunction and improve autophagic stress by increasing lysosome degradation.^17^, ^19^ Changes in lysosomal acidification and membrane permeability after AGE exposure are associated with lysosomal‐mitochondrial axis dysfunction and stress‐induced premature senescence in diabetic kidneys.^94^ Lysosomal dysfunction may also be a consequence of mTOR activation and TFEB inhibition.^89^ In db/db mice and AGE‐induced podocytes, activated mTOR sequestered TFEB in the cytosol, preventing it binding to the promoters of lysosomal target genes and decreasing their transcription.^95^ Together, these results suggest that AGEs, a major aetiological factor in DKD, can directly trigger lysosomal dysfunction and block downstream autophagic processes, thereby aggravating kidney lesions.

Nevertheless, a few studies have shown that AGEs increase the autophagosome markers in mesangial cells.^96^, ^97^ Inhibition of autophagy by cell‐specific deletion of *Atg5* could significantly aggravate the cytotoxic effects of AGEs in mesangial cells, indicating that autophagy activation plays a renoprotective role against AGE‐induced mesangial cell injury.^98^ However, most of these studies are based on the increase in expression of Atg, and the number of autophagic vacuoles and/or an accumulation of the autophagy substrate p62. Therefore, increased autophagosome number does not definitively indicate increased autophagy flux and may be due to blocked lysosomal trafficking. Moreover, this difference may be related to the experimental conditions such as the type of renal cells used and the purity or quality of fermented AGEs. Further studies are required to determine the specific effects of AGEs on the autophagic‐lysosomal process.

### 4.3 Urinary protein overload activates autophagy and triggers lysosome damage

Proteinuria is considered a major feature of DKD and a causative/aggravating factor of progressive renal injury. In PTECs, reabsorbed albumin is degraded in lysosomes, with excess albuminuria contained in glomerular filtrates imposing a large burden on the PTECs.^99^ The essential roles of autophagy in proteinuria‐induced kidney lesions have been elucidated in PTECs and podocytes. In PTECs, urinary proteins exert cytotoxic effects such as elevated ROS production, impaired mitochondrial function and endoplasmic reticulum stress, which may trigger autophagy to remove damaged organelles or misfolded proteins.^100^, ^101^ Our previous study also found an increase in the number of autophagic vacuoles in PTECs from patients with proteinuria nephropathy and rat models with severe proteinuria. Moreover, short‐term exposure to urinary proteins increased autophagic flux, as assessed by LC3‐II transit using a lysosomal inhibitor, suggesting that autophagic vacuole accumulation was caused by increased autophagosome formation in renal tubules.^102^ Consistently, inducing autophagic flux in PTECs by rapamycin treatment may protect cells from damage induced by urinary proteins,^103^, ^104^ while proteinuria‐induced damage is exacerbated in renal tubule‐specific Atg5 null mice, indicating that autophagy activation is crucial for protecting PTECs against tubular damage caused by urinary albumin.^105^ Thus, these data suggest that autophagy activation may act as a renoprotective mechanism against urinary proteins.

However, with prolonged proteinuria stimulation under diabetic conditions, impaired autophagy and damaged lysosomes may be involved in kidney injury induced by urinary proteins. Aberrant accumulation of p62 protein has been reported in the glomeruli of diabetic patients with massive proteinuria, suggesting impairment of the autophagy‐lysosomal pathway. Tagawa et al^12^ observed the accumulation of huge lysosomes with an increase in lamp2‐positive areas alongside an absence of autophagosomes in podocytes from massively proteinuric and diabetic rats and Atg5 null mice, suggesting that impaired autophagy due to urinary protein overload in podocytes is associated with lysosome dysfunction. Moreover, increased urinary proteins in glomerular filtrates during proteinuria may overload lysosomal pathways and cause lysosomal rupture in tubular cells, leading to renal tubular injury.^106^ Our previous study revealed that long time exposure to overloaded urinary protein induced deformed lysosomes and a decrease in lysosomal degradative ability, which may be the key node in the inhibition of autophagy in PTECs.^66^ Consistently, it was shown that lysosome number and volume increased in the PTECs of albumin‐overload rats due to lysosomal rupture and insufficient degradation,^100^ with increased lysosomal proteolysis counteracting protein accumulation in PTECs under proteinuria.^107^ Moreover, exposing renal tubules to excess albumin impaired autophagic flux in PTECs in a concentration‐dependent manner and significantly reduced the removal of long‐lived proteins via an mTOR‐mediated mechanism, suggesting inadequate lysosomal degradation.^108^ Furthermore, persistent and excessive albumin levels are found to down‐regulate Rab7 expression in PTECs and thus impair lysosomal function and autophagosome‐lysosome fusion.^109^ Together, these studies suggest that sustained exposure to urinary protein overload causes lysosome destabilization and dysfunction, and weakens autophagic degradation, while the pathological impairment of lysosomal function alongside normal autophagy induction sensitizes renal cells to autophagic stress.

Thus, short‐term stimulation with urinary proteins induces autophagy to remove protein aggregates and damaged organelles for renoprotection; whereas long‐term exposure obstructs lysosome‐mediated degradation and hence disrupts the renoprotective role of autophagy. Under these conditions, increased autophagy demand with impaired degradation in renal cells increase susceptibility to autophagic stress, causing renal cell death and exacerbating proteinuria. Therefore, stimulating autophagosome formation in renal cells may not be beneficial unless lysosomal degradation is restored.

### 4.4 Excessive ROS induce autophagy and cause lysosomal dysfunction

Persistent high glucose concentrations cause imbalance between ROS generation and antioxidant defences in kidneys, leading to oxidative stress that damages cellular constituents, including proteins, lipids and DNA.^110^ Accumulating data have suggested that ROS play an essential role in stress‐induced autophagy.^111^ Conversely, mitophagy can scavenge damaged mitochondria to remove oxidized proteins or damaged organelles and indirectly maintain kidney redox homeostasis. A number of studies have showed that oxidative stress involves various autophagy stimuli related to DKD, such as urinary protein, angiotensin II, palmitic acid (PA) or aldosterone related to DKD.^112^, ^113^ Nuclear ROS accumulation increases the synthesis of the nuclear factor (erythroid‐derived 2)‐like 2 (NRF2), hypoxia‐inducible factor 1(HIF‐1) and FOXO3, which stimulate the transcription of BNIP3 and p62.^114^ Additionally, increased ROS levels promote autophagy by activating lysosomal MCOLN1/TRPML1 channels to induce lysosomal Ca^2+^ release and trigger PPP3/calcineurin‐dependent TFEB nuclear translocation.^115^ Collectively, autophagy induction under mild oxidative stress stimulation seems to act primarily as a pro‐survival mechanism to protect against oxidative damage.

Conversely, excessive ROS production in the kidney under stress conditions is detrimental to lysosome function and autophagosome clearance. Previously, we showed that lysosomal lesions are related to LMP, as mediated by excessive ROS after urinary proteins or AGE exposure.^17^, ^18^, ^66^ Furthermore, the antioxidant vitamin E improves lysosomal function and increases autophagosome degradation, indicating that the evoked oxidative stress is closely associated with lysosomal impairment in diabetic kidneys.^19^ Similar results were observed in H_2_O_2_‐treated cells, with ROS production causing LMP and lysosomal Zn^2+^‐mediated mitochondrial fragmentation by stimulating TRPM2‐mediated extracellular Ca^2+^ entry.^63^ Accumulating evidence has suggested that lysosomal iron deposition in PTECs is a common pathogenic feature of early DKD,^116^ with dramatic increases in lysosomal redox‐active iron causing lysosomal membrane injury in renal cells by generating ROS.^117^ If robust oxidative stress leads to lysosomal membrane peroxidation, Fenton‐type reactions occur, resulting in lysosomal rupture that releases powerful proteolytic enzymes.^118^ The role of ROS overproduction in lysosomal dysfunction and autophagy impairment has also been determined in H_2_O_2_‐induced senescent cells, in which mitochondrial dysfunction and excessive ROS generation occur earlier than lysosomal dysfunction after H_2_O_2_ treatment.^119^ Thus, impairment of autophagic flux induced by sustained/excessive oxidative stress is associated with reduced lysosomal degradation and the intracellular accumulation of damaged organelles and protein aggregates within cells, which is a hallmark of accelerated kidney ageing in diabetes.

Collectively, a mild increase in ROS levels can signal both autophagy induction and lysosome biogenesis, thereby promoting autophagic flux, which may act as a pro‐survival signal. However, a large increase in ROS levels may trigger LMP, lysosomal dysfunction, and block autophagic flux as a pro‐death signal. Therefore, with supra‐threshold ROS stimulation in diabetic kidneys, the sustained high demand for autophagosome formation accompanied with gradually impaired lysosomal degradative function lead to autophagic stress, which is likely an important mechanism underlying the progression of DKD.

### 4.5 Lipid overload activates autophagy and impairs lysosomal function

Altered kidney lipid homeostasis has recently garnered increasing attention as an important detrimental factor in DKD progression,^120^ while lipotoxicity due to the accumulation of ectopic lipids has been associated with greater kidney damage susceptibility under hyperglycaemic conditions.^121^ Lipophagy is a form of selective autophagy that clears lipid deposits in diabetic kidneys.^26^ A high‐fat diet (HFD) or obesity has been reported to increase autophagy and protect against cytotoxicity during the progression of diabetes and kidney diseases.^122^, ^123^ Similarly, PA stimulation increases autophagosomes and autolysosome formation in podocytes by generating ROS against PA‐induced podocyte apoptosis.^113^ Moreover, lipid‐mediated alterations may also regulate autophagic flux in a time‐dependent manner, with short‐term PA overload shown to activate autophagy in the kidneys and redistribute membrane phospholipids into lysosomes to counteract renal lipotoxicity.^124^ Conversely, long‐term lipid overload impairs autophagy and causes excessive lipid accumulation in PTECs.

Emerging evidence has suggested that prolonged lipid overload may stagnate autophagic flux via mechanisms that impair lysosomal function in the kidney and exacerbate renal lipotoxicity. Mice given HFD exhibited proximal tubule injury with an increase of lipid vacuoles that were colocalized with enlarged LAMP1‐positive lysosomes, suggesting a dysfunction of the lysosomal system.^125^ The role of autophagy in obesity was further studied in PTECs where feeding HFD for 12 weeks resulted in autolysosomal dysfunction, accompanied by glomerular hypertrophy with mesangial matrix expansion.^126^ Moreover, Yamamoto et al^124^ investigated the HFD‐mediated autophagy changes in vivo using a pH‐sensitive probe, observing progressive autophagy stagnation during lipid overload due to impaired lysosomal acidification and thus excessive phospholipid accumulation. Indeed, fatty acids down‐regulate autophagy by reducing autophagosome‐lysosome fusion,^127^ as well as by decreasing the number and the acidity of lysosomes.^128^ Lipid overload impairs lysosomal function via various mechanisms; for instance, HFD‐mediated changes in lipid metabolism impair lysosomal formation by altering autophagosome and lysosome membrane composition. Conversely, long‐term lipid overload reduces mitochondrial function and triggers ATP depletion in PTECs, which may reduce lysosomal acidification and autophagic activity.^129^ Lipid overload may also diminish autophagic turnover via protein kinase C β (PKCβ)‐mediated NADPH oxidase (Nox)2 activation, which generates ROS that impair lysosomal acidification and pH‐dependent lysosomal enzyme activity.^130^ HFD‐induced autolysosomal dysfunction is caused primarily by megalin‐mediated endocytosis, which may affect the handling of toxic glomerular‐filtered lipotoxic substances and thus endosome and lysosome function in PTECs.^126^ In addition, high lipid levels lead to constant mTOR activation and alter lysosomal adaptation via TFEB and TFE3, dysregulating lipid and glucose homeostasis throughout the body.^131^ Our understanding of lysosomal function as a nutrient signalling hub has greatly increased recently; however, further studies are required to elucidate the mechanisms via which lipids regulate the lysosome in diabetic kidneys.

In summary, lipid overload effectively induces autophagy to maintain lipid homeostasis, which exerts a crucial role in counteracts renal lipotoxicity during DKD; however, sustained lipid overload, and subsequent autophagic activation, places a huge burden on the lysosomal system, resulting in lysosomal dysfunction. Increased or sustained high autophagy demand in the face of impaired lysosomal degradation, contributes to autophagic stress, a process related to DKD development. Thus, due to its regulatory effects on lipid metabolism, lipophagy could be a novel therapeutic target for DKD and warrants further investigation.

## 5 POTENTIAL TARGETS FOR IMPROVING AUTOPHAGIC STRESS

Developing DKD treatments requires an understanding of the specific stages of autophagy altered in DKD; for instance, during autophagy dysregulation due to lysosomal dysfunction, further autophagosome formation may aggravate homeostatic perturbations. Instead, interventions that enhance lysosomal degradation may relieve blockages by removing non‐functional substrates to yield therapeutic effects, as could the proper inhibition of autophagy up‐regulation.^132^ Thus, restoring the balance between autophagosome formation and lysosomal degradation should be beneficial, regardless of the underlying cause. In DKD, strategies that accelerate downstream degradation of autophagy by reducing lysosomal damage or promoting lysosomal biogenesis may be promising novel therapies to increase overall autophagy activity (Table 1).

**TABLE 1:** Summary of strategies aimed at accelerating downstream degradation of autophagy in DKD

Compound examples	Disease model	Effects on lysosomal function and biogenesis	References
Antioxidant N‐acetylcysteine or catalase	AGEs‐induced human PTECs (HK‐2 cells)	Improving the lysosomal acidification and degradation	[17]
a‐tocopherol	Streptozotocin (STZ)‐induced diabetic rats and AGEs‐induced HK‐2 cells	Increasing lysosomal enzymatic activity and lysosomal degradation	[19]
Resveratrol plus a‐tocopherol	AGEs‐induced mouse podocytes	Increasing lysosomal enzymatic activity and lysosomal degradation	[18]
Curcumin and quercetin	STZ‐induced diabetic rats	Increasing lysosomal enzymatic activity	[81]
mTOR inhibitor, Torin1	Db/db mice; AGE‐stimulated mouse podocytes	Promoting nuclear TFEB expression.	[95]
Histone deacetylase 6 (HDAC6) inhibitor, Tubastatin A	Subtotally nephrectomized rats and NRK‐52E cells	Increasing TFEB acetylation and enhancing the nuclear localization of TFEB	[16]

One strategy for lysosomal restoration is improving the stability of lysosomal structure and function. For example, the heat‐shock protein‐70 (Hsp70) can stabilize protective lysosomal proteins, and safeguard lysosomal membranes against LMP induced by various stimuli,^133^, ^134^ while its pharmacological induction has been reported to improve renal function in diabetic kidneys.^135^, ^136^ In addition, given the principal role of ROS in triggering LMP, antioxidants such as N‐acetylcysteine and a‐tocopherol may protect renal cells from LMP and apoptotic cell death.^137^, ^138^ Recently, we showed that the typical antioxidant vitamin E has the protective effect on lysosomes to reduce the autophagic stress.^19^ Notably, in recent years plant‐based flavonoids such as resveratrol and quercetin also exert antioxidant effects and have attracted attention in terms of their potential use as lysosomal protectors against oxidative aggression.^139^, ^140^ Although many DKD pathogenic factors trigger LMP, few attempts have been made to pharmacologically improve this process.

The other strategy is the transcriptional induction of genes for the biogenesis of new lysosomes to compensate for the loss of lysosomal degradative capacity.^141^ For instance, enhancing TFEB nuclear translocation to increase lysosomal biogenesis is one of the most promising strategies for improving lysosomal function.^141^, ^142^ Stimulating TFEB expression increases the clearance of toxic proteins in lysosome‐related diseases and may be renoprotective in DKD.^16^, ^143^ The majority of known TFEB activators are mTOR inhibitors^95^; however, mTOR‐independent TFEB activators may cause less cellular damage. Recent evidence has suggested that the natural disaccharide trehalose restores autophagic flux via mTOR‐independent TFEB activation^144^, ^145^ and exerts cytoprotective effects in renal cells, potentially by improving lysosomal alkalization and dysfunction.^146^ In addition, polyphenolic compounds, such as such as curcumin, quercetin and genistein, have been discovered to be able to stimulate endogenous TFEB activity, thereby providing an attractive alternative to gene‐transfer therapy in lysosomal‐related diseases.^147^, ^148^, ^149^ Curcumin, the active ingredient in Curcuma longa, promotes TFEB nuclear translocation and activates lysosomal function, as evidenced by the increase in lysosomal acidification and enzyme activity, thus enhancing autophagic flux.^149^ While several proof‐of‐concept studies have shown the beneficial effects, further studies are required to explore pharmacological modulation with available drugs to promote lysosomal biogenesis in DKD.

## 6 CONCLUSIONS AND FUTURE PERSPECTIVES

Metabolic stresses in diabetes, such as hyperglycaemia, urinary proteins, mild oxidative stress and dyslipidaemia, activate the autophagy‐dependent turnover of damaged cellular constituents to maintain cellular homeostasis and ultimately protect nephrons against injury. However, prolonged and intense stresses accompanied by autophagic activation inevitably burden the lysosomal system and progressively stagnate autophagic flux, increasing vulnerability to additional stress and aggravating kidney dysfunction. Thus, with alterations in the lysosomal homeostasis under chronic hyperglycaemia, AGE generation, massive proteinuria, excessive ROS and lipid overload reduce normal or enhanced autophagic activation and cause autophagic stress, which activates damage‐response mechanisms involved in DKD pathogenesis. Autophagic stress due to lysosomal dysfunction in diabetic kidneys may reconcile the conflicting roles of activated autophagy in DKD; thus, restoring or augmenting lysosomal degradation to remove blockages in the autophagic‐lysosome process could provide promising therapies for DKD. In most cases, processes upstream of autophagy, such Atg expression and autophagosome number, have been predominantly studied in renal intrinsic cells, which are not sufficient for evaluating autophagy activity. Downstream processes should therefore be studied to improve our understanding of autophagy in DKD progression, including autolysosome efficiency, lysosomal enzyme activity, autophagic vacuole degradation and lysosomal biogenesis. Our knowledge of druggable autophagy targets in DKD remains limited, and current autophagy assays pose challenges for effective implementation. Advances in big data, multiomics studies and artificial intelligence may help to identify pharmacodynamic markers in the autophagic‐lysosomal pathway and integrate mechanistic drug discovery data.^150^

## CONFLICTS OF INTEREST

All authors declare that they have no conflicts of interest.

## AUTHOR CONTRIBUTIONS

WJL and YXW developed and organized this paper. HJZ and XQZ mainly drafted the paper and created the figures. All other authors participated in revising the paper and finalizing the manuscript. All authors gave approval for publication.

## Data Availability

All data generated or analysed during this study are included in this article.
